# Radioactivity levels in the saliva of patients undergoing targeted radioligand therapy with [^177^Lu]Lu-PSMA-I&T and [^177^Lu]Lu-DOTA-TOC

**DOI:** 10.1007/s00784-025-06300-w

**Published:** 2025-04-11

**Authors:** Christian Schedeit, Nasir Gözlügöl, Radi Saiyed Alsheikh, Mohamed Shelan, Robert Seifert, Federico Caobelli, Urs Borner, Tateyuki Iizuka, Benoît Schaller, Axel Rominger, Paul Cumming, Ali Afshar-Oromieh, Konstantinos G. Zeimpekis

**Affiliations:** 1 Department of Cranio-Maxillofacial Surgery, Bern University Hospital, Inselspital, University of Bern, Bern, CH-3010 Switzerland; 2 Department of Nuclear Medicine, Bern University Hospital, Inselspital, University of Bern, Freiburgstr. 18, Bern, CH- 3010 Switzerland; 3 Department of Radiation Oncology, Inselspital Bern, University of Bern, Bern, Switzerland; 4 Department of Otorhinolaryngology, Head and Neck Surgery, Inselspital, University Hospital, University of Bern, Bern, Switzerland; 5 School of Psychology and Counselling, Queensland University of Technology, Brisbane, Australia

**Keywords:** Saliva, Radionuclide therapy, Prostate cancer, PSMA, 177Lu-PSMA, MRONJ

## Abstract

**Objectives:**

The number of patients receiving radioligand therapy (RLT) has risen sharply in recent years. This raises concerns about possible risks to dental healthcare workers due to their exposure to the patients and their saliva. We therefore set about to measure the salivary radioactivity in patients undergoing ^177^Lu–RLT.

**Materials and methods:**

We recruited in-house RLT patients receiving [^177^Lu]Lu -DOTA-TOC (*n* = 6) or [^177^Lu]Lu-PSMA-I&T (*n* = 14). We measured the radioactivity concentrations in 1 ml saliva samples collected before and 0.5, 2, 4, 21, 27, and 45 h post application of the radioligands, with additional samples collected at 51 and 69 h for [^177^Lu]Lu-PSMA-I&T patients. The biological half-life (BHL) and area under the curve (AUC) were calculated for the radioactivity of the saliva for both cohorts.

**Results:**

Both cohorts exhibited increases in salivary radioactivity, attaining peaks at 2 h p.i. of [^177^Lu]Lu-DOTA-TOC and 4 h p.i. of [^177^Lu]Lu-PSMA-I&T, and presenting with a significant decrease until the patients discharge. The median peak concentration for [^177^Lu]Lu-PSMA-I&T was four-fold higher than for the [^177^Lu]Lu-DOTA-TOC group. For PSMA-patients, the BHL was 14 h and the mean AUC was 895 kBqh/ml. For DOTA-TOC patients, these values were 8.5 h and 96 kBqh/ml, respectively.

**Conclusion:**

Salivary radioactivity peaks earlier and at lower levels in [^177^Lu]Lu-DOTA-TOC patients compared to [^177^Lu]Lu-PSMA-I&T, which shows longer retention and ten times higher radioactivity turnover in saliva. However, radiation exposure to medical staff by the patents saliva can be considered minimal.

**Clinical relevance:**

Salivary radioactivity of patients undergoing ^177^Lu-RLT poses minimal risk to oral healthcare workers.

## Introduction

Surgical resection remains the treatment of choice for non-metastatic neuroendocrine tumors (NET) and prostate cancer (PCa). However, with the introduction of targeted radioligand therapy (RLT), the management of metastatic NET and PCa has been transformed [1–5]. In contrast to conventional chemotherapy and radiotherapy, targeted RLT utilizes radiolabeled drugs, peptides, or antibodies to deliver ionizing radiation specifically to tumor sites, taking advantage of the binding properties of the carrier molecules. Imaging for NET began in the 1990s with the deployment of the somatostatin analog Indium-111-labeled octreotide (^111^In-octreotide). ^111^In decays via electron capture by emitting gamma rays of 171 keV and 275 keV. Today, targeted RLT for NET is primarily based on Lutetium-177-octreotate/octreotide ligands, such as [^177^Lu]Lu-DOTA-TOC [6]. While these RLT molecules show minimal physiological uptake in the salivary glands [7], prostate-specific membrane antigen (PSMA)-targeted ^177^Lu-radioligands [8] show extensive accumulation in healthy salivary glands [9]. Unintended irradiation of the salivary glands is a frequent cause of xerostomia in metastatic PCa patients undergoing multiple cycles of [^177^Lu]Lu-PSMA-I&T [10].

The mechanism behind the uptake and retention of [^177^Lu]Lu-PSMA-I&T in the salivary glands remains uncertain, and it is not clearly established whether this RLT conveys significant amounts of radioactivity into the patients’ saliva. Little is known about the potential health risks posed to healthcare providers and PCa patients from salivary secretion of radioactivity during a course of [^177^Lu]Lu-PSMA-I&T RLT. Dental hygienists, dentists, otolaryngologists, plastic surgeons, and maxillofacial surgeons may be called upon to treat such patients and, in doing so, could incur radiation exposure. Additionally, PCa patients undergoing treatment may be vulnerable to the detrimental effects of buccal radioactivity, particularly those receiving concomitant bone-protective therapy. The frequent occurrence of bone metastases in castration-resistant prostate cancer (CRPC) patients increases the risk of spinal cord compression and disabling fractures. Antiresorptive therapy with the monoclonal antibody Denosumab or the bisphosphonate zoledronic acid can significantly reduce these events [11], but at the risk of medication-related osteonecrosis of the jaw (MRONJ) [12].

Given these considerations, we aimed to assess radiation levels in the saliva of PCa patients undergoing a cycle of [^177^Lu]Lu-PSMA-I&T RLT, in order to evaluate potential risks to healthcare providers and patients predisposed to osteonecrosis of the jaw. We also examined NET patients treated with [^177^Lu]Lu-DOTA-TOC to measure and compare the radioactivity levels in saliva across both groups.

## Materials & methods

### Patient population

This study was a prospective cohort study including 20 consecutive patients with NET or PCa who underwent targeted radioligand therapy at the Department of Nuclear Medicine, Inselspital, Bern University Hospital, from November to December 2023. The local ethics committee approved the study (2023 − 01354) and all participants provided signed informed consent. The prospective cohort consisted of six patients treated with [^177^Lu]Lu-DOTA-TOC for metastatic NET at different primary sites, and 14 patients treated with [^177^Lu]Lu-PSMA-I&T for metastatic PCa. In both groups, the tumors were considered inoperable, and all first- and second-line therapies had been exhausted, meaning that RLT was administered palliatively. All treatments were conducted in accordance with the guidelines of the European Neuroendocrine Tumor Society, the Oncology Guideline Program of the Association of the Scientific Medical Societies in Germany (AWMF), the German Cancer Society (DKG), the German Cancer Aid Foundation (DKH), and Swiss national regulations. For each RLT course, patients were hospitalized for two consecutive days for [^177^Lu]Lu-DOTA-TOC and three consecutive days for [^177^Lu]Lu-PSMA-I&T.

### Study design

The primary endpoint was the absolute radioactivity concentration in series of saliva samples collected from patients treated with [^177^Lu]Lu-PSMA-I&T or [^177^Lu]Lu-DOTA-TOC, as described below. The secondary endpoints included changes in radioactivity concentration following the administration of ^177^Lu-labeled pharmaceuticals, the time at which radioactivity concentration reaches its peak, the general time-dependent course of radioactivity in saliva for both groups and potential exposure for dentists and medical staff.

### Measurement with a gamma counter

Saliva samples were collected at the predefined time intervals: background samples just prior to RLT and at 30 min, 2, 4, 21, 27, 45 h post-injection for NET/[^177^Lu]Lu-DOTA-TOC patients. For the PCa/[^177^Lu]Lu-PSMA-I&T patients, additional samples were collected at 51 and 69 h due to the longer hospital stays of these patients. RLT administrations were performed according to the daily work routines at the therapy ward. Patients were asked to provide saliva samples of approximately 2–3 mL by expectoration into disposable 10 ml sputum containers. Afterward, using a micropipette, a standard volume of exactly 1 ml was extracted from each saliva sample and placed into a Radioimmunoassay (Ria) polystyrene (PS) tube. The samples were then measured in a WIZARD^2^ 2740 automatic gamma counter (PerkinElmer Inc., Waltham, MA, USA), with the energy window set to 113 keV ± 10% (range 111–124 keV).

Before the salivary measurements, we calibrated the gamma counter using triplicate sample tubes, each containing 500 Bq/ml of ^177^Lu, (t_1/2_ 6.7 days). The background radiation were measured in three 1mL water samples, and the mean of these values was subtracted. The final calibration factor was determined to be 1841 counts per minute per kilobecquerel (cpm/kBq).

### Biological half-life and area under the curve (AUC)

The biological half-life (BHL) was calculated from the linear-fitting slope of the late phase of the saliva’s radioactivity concentration, plotted on a logarithmic scale for all patients. We then determined the medians for both cohorts. Additionally, we calculated the area under the curve (AUC) for all time points in both cohorts to quantify the total salivary radioactivity in kBq·h/ml.

### Statistical analysis

For data analysis, we used SPSS Statistics version 22 (IBM Corp. Released 2013. IBM SPSS Statistics for Windows, Version 22.0. Armonk, NY: IBM Corp.). A descriptive analysis was conducted for relative frequencies, means, and their corresponding standard deviations or medians. Time series of mean salivary radioactivity from [^177^Lu]Lu-DOTA-TOC and [^177^Lu]Lu-PSMA-I&T were compared using the Mann-Whitney U test (Wilcoxon rank-sum test). P values < 0.05 were considered statistically significant.

## Results

### Patient characteristics

There were six NET/[^177^Lu]Lu-DOTA-TOC patients (four males) and 14 PCa/[^177^Lu]Lu-PSMA-I&T patients. Both groups received similar injected activities, with some patients undergoing multiple cycles, particularly in the PSMA group, where one patient had undergone the 9th cycle. The interval between treatments was 8 weeks ± 1 week for [^177^Lu]Lu-DOTA-TOC and 6–7 weeks for [^177^Lu]Lu-PSMA-I&T. The number of treatment cycles received by each patient is listed in Table 1. The 14 PCa patients had a mean (SD) age of 71.6 ± 11.1 years (range 51 to 91 years) and the NET patients had a mean age of 58.0 ± 6.7 years (range 51 to 70 years; four males). The range of the injected activity for [^177^Lu]Lu-DOTA-TOC was 7.3–7.7 GBq, while for PSMA 6.9–7.5 GBq. The exact activities and patient demographics are provided in Table 1.

**Table 1:** Patient demographics including age, sex, the injected activity and the treatment cycle

Patient	Age	Sex	Radiopharmaceutical	Injected Activity (GBq)	Treatment Cycle
#1	61	M	177Lu-DOTATOC	7.4	#4
#2	55	F	177Lu-DOTATOC	7.7	#1
#3	70	M	177Lu-DOTATOC	7.3	#2
#4	15	M	177Lu-DOTATOC	7.5	#3
#5	56	F	177Lu-DOTATOC	7.5	#2
#6	55	M	177Lu-DOTATOC	7.6	#3
#7	65	M	177Lu-PSMA I&T	7.5	#1
#8	73	M	177Lu-PSMA I&T	7.5	#1
#9	70	M	177Lu-PSMA I&T	7.4	#3
#10	63	M	177Lu-PSMA I&T	7.3	#2
#11	68	M	177Lu-PSMA I&T	7.4	#4
#12	77	M	177Lu-PSMA I&T	7.4	#2
#13	51	M	177Lu-PSMA I&T	7.2	#2
#14	72	M	177Lu-PSMA I&T	7	#3
#15	75	M	177Lu-PSMA I&T	6.9	#4
#16	88	M	177Lu-PSMA I&T	7.3	#5
#17	55	M	177Lu-PSMA I&T	7.3	#3
#18	77	M	177Lu-PSMA I&T	7.1	#1
#19	91	M	177Lu-PSMA I&T	7.3	#3
#20	78	M	177Lu-PSMA I&T	7.4	#9

### Primary endpoint

**Fig. 1:**
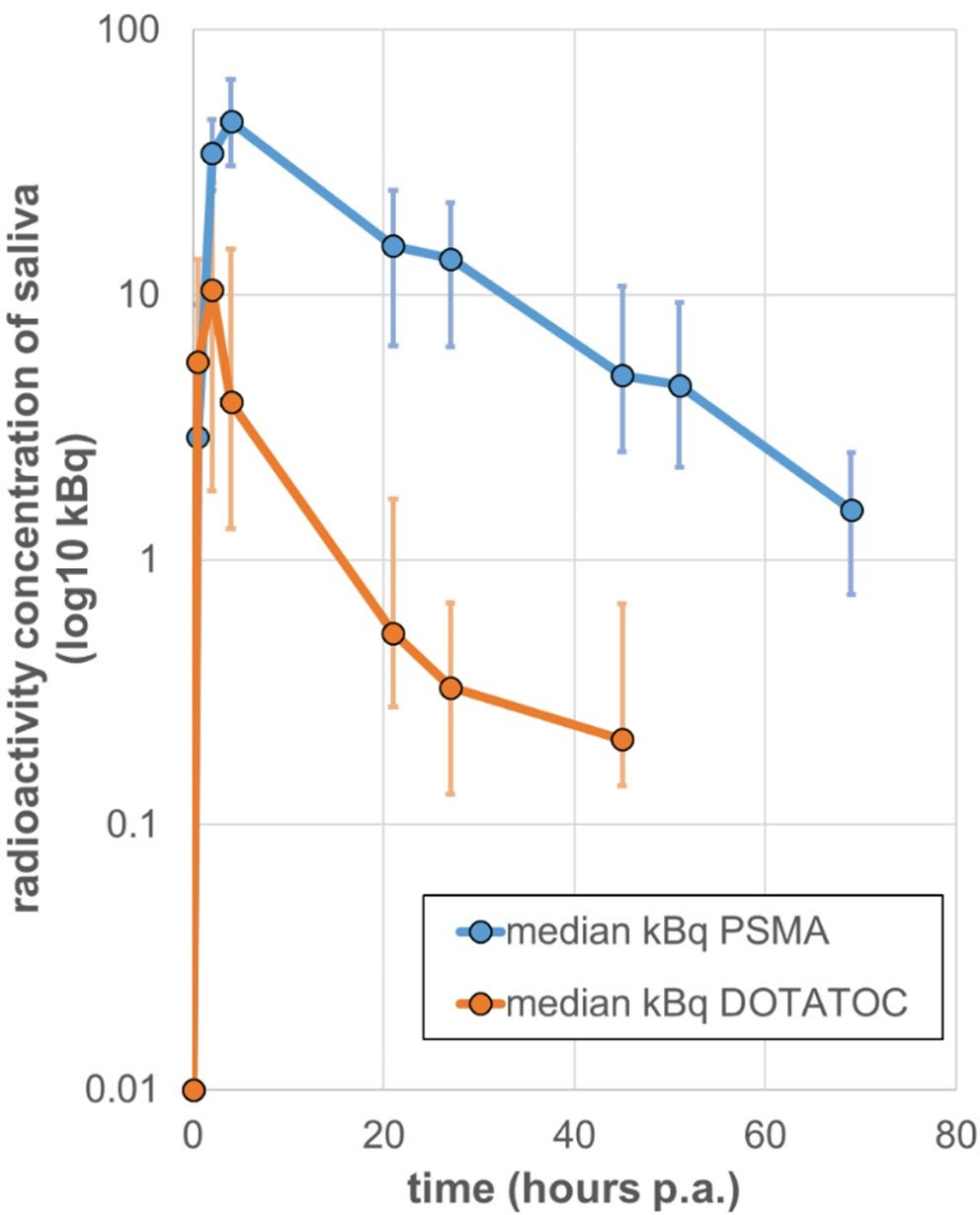
Median salivary radioactivity concentrations (log_10_ kBq/ml) with corresponding interquartile range (IQR) bars, shown in logarithmic scale as a function of time post-application (p.a.) of [^177^Lu]Lu-DOTA-TOC (*n* = 6; orange curve) and [^177^Lu]Lu-PSMA-I&T (*n* = 14; blue curve)

All RLT patients showed significant salivary radioactivity, with peak concentrations observed at 2 h post injection (p.i.) for [^177^Lu]Lu-DOTA-TOC and at 4 h p.i. for [^177^Lu]Lu-PSMA-I&T, followed by a continuous decline thereafter (Fig. 1). The pretreatment saliva measurements confirmed the absence of remnant radioactivity from the previous treatment cycle in each individual. The median peak saliva concentrations were 10 kBq/ml at 2 h for [^177^Lu]Lu-DOTA-TOC and 45 kBq/ml at 4 h for [^177^Lu]Lu-PSMA-I&T. Complete statistics are provided in Table 2.

**Table 2:** Statistical evaluation of both patient cohorts, for [^177^Lu]Lu-DOTA-TOC and [^177^Lu]Lu-PSMA-I&T radioactivity uptake in saliva, in terms of median and interquartile range (kBq) for all measurement time points

	[^177^Lu]Lu-DOTA-TOC(kBq/ml)		[^177^Lu]Lu-PSMA-I&T (kBq/ml)	
**time p.a. (h)**	median	interquartile range	median	interquartile range
0	0.01	0.01	0.01	0.01
0.5	5.58	10.70	2.92	7.50
2	10.42	24.81	34.19	20.93
4	3.94	13.51	45.12	34.46
21	0.53	1.42	15.3	18.29
27	0.33	0.56	13.69	15.81
45	0.21	0.54	4.96	8.20
51			4.56	7.08
69			1.55	1.80

In addition to differing peak times, the salivary radioactivity concentrations exhibited marked kinetic differences. Radioactivity from [^177^Lu]Lu-DOTA-TOC showed a plateau near the detection limit in samples collected at 21 and 27 h, with further decline in the last sample before discharge (i.e., 210 Bq/ml at 45 h). In contrast, radioactivity concentrations from [^177^Lu]Lu-PSMA-I&T were consistently more than three times higher than those from [^177^Lu]Lu-DOTA-TOC, remaining at 1.5 kBq/ml at 69 h.

The median biological half-life (BHL) was 14 h (range: 10–39 h) for [^177^Lu]Lu-PSMA-I&T and 8.5 h (range: 6–13 h) and [^177^Lu]Lu-DOTA-TOC patients. Furthermore, the median AUC was 96 kBq·h/ml for [^177^Lu]Lu-DOTA-TOC and 895 kBq·h/ml for [^177^Lu]Lu-PSMA-I&T patients. The Mann-Whitney U-test showed that salivary radioactivity concentrations from [^177^Lu]Lu-PSMA-I&T exceeded those from [^177^Lu]Lu-DOTA-TOC at 2 h after the administration (*p* < 0.05), and remained consistently higher thereafter. In addition, radioactivity from [^177^Lu]Lu-DOTA-TOC reached a faster peak in saliva, but showed faster clearance.

## Discussion

RLT with [^177^Lu]Lu-DOTA-TOC is a well-established treatment for metastatic neuroendocrine tumors (NET), particularly in cases where conventional therapies have failed. This treatment aims to slow tumor progression and improve patient outcomes, though it can also be used palliatively in patients with advanced disease to alleviate symptoms and improve quality of life. The uptake of [^177^Lu]Lu-DOTA-TOC in salivary glands is low, resulting in lower associated risk for radiation-induced damage to the oral cavity. The number of NET patients is significantly lower compared to PCa patients. Moreover, the latter are often treated with osteoprotective medications in the advanced stages of the disease, which in turn carry a risk of jaw necrosis. Furthermore, our results have shown that the amount of radioactivity in the saliva of NET patients undergoing RLT is negligibly low. For these reasons, the following discussion focuses on PCa patients receiving ¹⁷⁷Lu-PSMA therapy.

Treatment with [^177^Lu]Lu-PSMA-ligands has become a widely used and increasingly common treatment modality for metastatic PCa. There is a need to understand the radiation-induced side effects of these radioligands and their potential interactions with other drugs. This is particularly relevant in vulnerable areas of the oral cavity, especially when combined with anti-resorptive drugs, which are commonly prescribed to patients with metastatic prostate cancer and are known to cause osteonecrosis of the jaw. In addition to the impact on patients, the potential effects of the radiation on doctors and medical staff are also of concern. To the best of our knowledge, no data have been published on the level of radioactivity in the saliva of patients undergoing [^177^Lu]Lu-RLT. Therefore, this study focused on measuring the radioactive content in saliva to assess its potential impact on the radiation exposure of dental professionals treating oral and jaw-related conditions in RLT patients.

Regarding radiation exposure to the hands of medical staff from direct contact with the saliva of RLT patients, measurements taken at discharge (48 h post-administration) indicate that even the highest recorded salivary radioactivity in a [^177^Lu]Lu-PSMA-I&T patient (~ 20 kBq) results in a dose rate of less than 2000 µSv/hr at a distance of 1 cm. For the median salivary radioactivity across all [^177^Lu]Lu-PSMA-I&T patients (~ 4.5 kBq), the estimated dose rate is four times lower, at 500 µSv/hr. Similarly, the dose rate for the maximum (~ 0.8 kBq) and median (~ 0.2 kBq) saliva uptake of [^177^Lu]Lu-DOTA-TOC patients at discharge is less than 100 µSv/hr for both. Therefore, the annual dose limit of 500 mSv for extremities will not be exceeded. By the time patients are discharged, saliva radioactivity levels have decreased, making the risk during subsequent care in the following days negligible or nonexistent. This is further supported by the lack of residual radioactivity in saliva samples at the start of subsequent treatment cycles.

Daily saliva production varies between 0.5 and 1.5 L [13]. In this study, the median peak radioactivity in saliva was 45 kBq/ml. Considering a daily production of 1000 ml saliva, the median total radioactivity amount in the saliva would be 45.0 MBq for patients receiving [^177^Lu]Lu-PSMA-I&T. A similar calculation can be made for patients receiving [^177^Lu]Lu-DOTA-TOC, whose median peak radioactivity was 10 kBq/ml. In this case, the median total radioactivity in the saliva would be 10 MBq.

This study observed a difference in radioactivity levels between the two treatment groups. Despite the higher uptake of [^177^Lu]Lu-PSMA-I&T in the salivary glands [14], it did not result in an equally higher radioactivity level in the saliva compared to [^177^Lu]Lu-DOTA-TOC. The trapping of radioactivity in the salivary glands has been previously described in the literature [15].

**Fig. 2:**
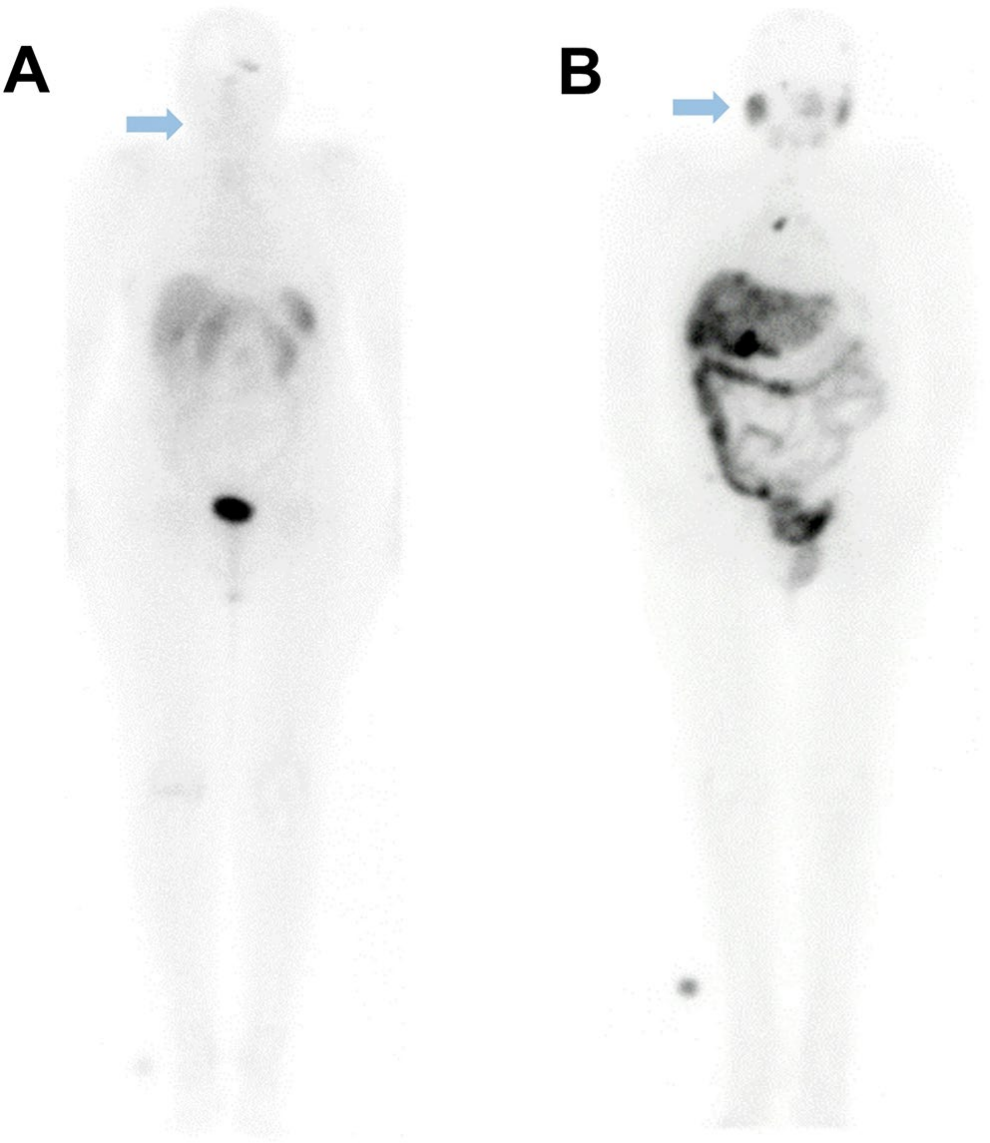
Planar imaging of representative patients during RLT with (**A**) [^177^Lu]Lu-DOTA-TOC and (**B**) [^177^Lu]Lu-PSMA-I&T at 24 h post-application. The arrows indicate the non-uptake and uptake of the radiopharmaceutical in the salivary glands, respectively

Patients treated with [^177^Lu]Lu-DOTA-TOC exhibited minimal uptake in the salivary glands (Fig. 2). However, some radioactivity was detected in the saliva, likely due to direct secretion from the capillaries of the oral mucosa. Although direct evidence for specific (receptor-mediated) uptake of DOTA-TOC in the salivary glands is lacking in the literature, our findings are consistent with those observed for DOTA-TATE, which has a similar biodistribution [16–18].

Our data also showed longer radioactivity retention in the saliva for [^177^Lu]Lu-PSMA-I&T compared to [^177^Lu]Lu-DOTA-TOC with a biological half-life of 14 h compared to 8.5 h, and a tenfold higher turnover activity accumulation in the saliva, based on the AUC calculation.

Concerning the whole-body radiation exposure of medical staff, other studies have shown the external dose rate from patients treated with [^177^Lu]Lu-DOTA-TATE or [^177^Lu]Lu-PSMA at various time points and distances [19–21]. These dose rates are very similar for both treatments at 48 h post-radiopharmaceutical administration at any given distance (from direct contact up to 2 m). At 25 and 50 cm, which can be assumed to be the typical distances between the patient and medical staff during a dental treatment, the dose rate at 48 h would be 21 µSv/hr and 10 µSv/hr, respectively.

A previous study documented that the dose rate from [^177^Lu]Lu-PSMA-I&T patients, five days after administration, was 11 µSv/hr at direct contact (compared to 536 µSv/hr immediately after administration) and 1 µSv/hr at a one-meter distance from the patient (48 µSv/hr immediately after administration) [21]. The estimated exposure of medical staff from the patient’s body ranged from 1 µSv/hr (dose rate at 1-meter distance) to 4 µSv/hr (projected dose rate at 0.5-meter distance). It is highly improbable that the annual dose limit of 1 mSv per year (1000 µSv) for personnel non-professionally exposed to ionizing radiation will be exceeded.

Xerostomia is one of the most common side effects of PSMA-RLT. Given the well-known sensitivity of the salivary glands to radiation-induced damage, it is evident that there is a need for a better understanding of the relationships in this area [22].

Patients with metastatic PCa are frequently treated with antiresorptive drugs, and additional systemic radiopharmaceuticals may cause unknown tissue reactions in the jaw. While MRONJ is generally rare, these patients face an increased risk, with recent studies reporting rates of 8.4% and 15% [12, 23–25].

In light of these findings, further research is needed to explore the long-term effects of radioligand therapy, particularly xerostomia and osteonecrosis of the jaw, especially since many patients have received antiresorptive therapies.

Our study has certain limitations: The small sample size may limit the generalizability of the results. Additionally, the biological half-lives and AUC values show considerable variability between patients, which could suggest inter-individual differences in the pharmacokinetics of the radioligands that cannot be fully explained. Moreover, the study makes assumptions about the radiation exposure of medical personnel based on dose measurements and theoretical modelling. Concrete long-term data on the actual exposure of personnel are lacking.

## Conclusion

This study demonstrates the excretion of radioactivity into saliva in patients undergoing [^177^Lu]Lu-PSMA-I&T and [^177^Lu]Lu-DOTA-TOC therapies, with peak levels occuring shortly after administration and rapidly decreasing thereafter. [^177^Lu]Lu-PSMA-I&T exhibits a longer biological half-life and a tenfold higher radioactivity concentration in saliva compared to [^177^Lu]Lu-DOTA-TOC. Two days after radiopharmaceutical administration, the potential radiation exposure risk to medical staff treating dental or oral issues in these patients is minimal, both for the whole-body effective dose and for hand exposure.

## Data Availability

The data are available in an anonymized Excel file upon request.
